# Differential analysis of splenic immune indicators, transcriptomic profiles, and metabolomic features in pigs under liquid–solid feeding conditions

**DOI:** 10.3389/fvets.2025.1735383

**Published:** 2026-01-13

**Authors:** Yangguang Liu, Cuiyun Zhu, Huibin Zhang, Fan Xie, Haibo Ye, Shiming Zhao, Qianqian Wang, Xianrui Zheng, Zongjun Yin, Xiaodong Zhang

**Affiliations:** 1 College of Animal Science and Technology, Anhui Agricultural University, Hefei, China; 2 Anhui Provincial Key Laboratory of Livestock and Poultry Product Safety, Institute of Animal Husbandry and Veterinary Medicine, Anhui Academy of Agricultural Sciences, Hefei, China

**Keywords:** fermented liquid feed, immunity, metabolome, pig, transcriptome

## Abstract

**Introduction:**

Fermented liquid feed (FLF) has been shown to improve feed efficiency and growth performance of pigs, however, its effects on porcine immune function remain poorly understood.

**Methods:**

In this study, transcriptomic, metabolomic, and ELISA-based approaches were employed to systematically evaluate the effects of fermented liquid feed on immune factor levels, splenic metabolic profiles, and gene expression in pigs. A total of 64 commercial pigs were randomly assigned to two groups, fed a basal diet group (CON) and a fermented liquid diet group (LFF), for a feeding period of 60 days.

**Results:**

The results showed that, compared with the CON group, the LFF group exhibited significantly higher serum concentrations of interleukin-6(IL-6, 767.88 ± 12.43 pg/ml), tumor necrosis factor-α(TNF-α 678.32 ± 15.37 pg/ml), complement C3(145.92±3.69 μg/ml), immunoglobulin A(IgA, 485.15 ± 9.13 μg/ml), and interferon-γ(IFN-γ 1966.76 ± 72.22 pg/ml). Meanwhile, transcriptome sequencing revealed that the expression levels of immune-related genes *CXCL2*, *CXCL8*, and *SLA-5* were significantly upregulated in the LFF group. Further KEGG pathway analysis demonstrated a more pronounced enrichment of the chemokine signaling pathway and IL-17 signaling pathway in this group. The metabolomic analysis revealed that fermented liquid feed altered the metabolic profile of the spleen, primarily affecting the biosynthesis of unsaturated fatty acids and linoleic acid metabolism. Integrated analysis indicated that fermented liquid feed reprogrammed metabolic patterns by modulating the expression of immune-related genes.

**Discussion:**

In conclusion, our findings indicate that fermented liquid feed significantly enhances immune function in pigs, providing a theoretical basis for its scientific application and promotion in healthy farming of pigs.

## 1 Introduction

Fermented liquid feed (FLF) typically refers to a mixed feed prepared by combining feed and water at a ratio of 1:1.5 to 1:4, followed by anaerobic fermentation mediated by naturally occurring or inoculated microorganisms(such as lactic acid bacteria and yeasts) (1). During fermentation, microorganisms can degrade anti-nutritional factors and macromolecular nutrients in the feed, including cellulose, lignin, and proteins, thereby improving nutrient bioavailability and the overall nutritional value of the feed (2, 3). Zhang et al. reported that probiotic-fermented feed markedly reduced antinutritional factors and biogenic amines, while concurrently enhancing crude protein, acid-soluble protein, enzymatic activity, and antioxidant capacity in complete pig feed (4). Recent studies indicate that amino acid loss occurs in fermented liquid feed, leading to reduced feed efficiency (5). However, another report suggests that fermented liquid feed can increase the abundance of beneficial bacteria in the gut and enhance growth performance (6). Furthermore, fermented liquid feed can also influence meat quality traits by promoting muscle fiber growth and regulating meat color through adipocytokine signaling pathways (7).

In recent years, the European Union has banned the use of antibiotics as growth promoters in animal production within Europe (8), there has been an urgent need for safe and effective alternatives (9). Fermented liquid feed can provide animals with probiotic and improve growth performance, and thus has the potential to serve as an effective feeding strategy to replace antibiotic growth promoters (10). Research indicates that during the fattening phase of pig production, feeding fermented liquid feed can modulate the gut microbiota, increase the production of lactic acid and acetic acid, lower intestinal pH, and ultimately enhance intestinal health (11). Further research has indicated that fermented feed can increase the abundance of immune-related metabolites in the rumen of ruminants, activate multiple immune pathways including the IL-17 signaling pathway and the NOD-like receptor signaling pathway, and simultaneously regulate various immune processes such as T cell migration and chemokine production (12, 13). Therefore, in-depth investigation into the mechanisms by which FLF modulates host immune function may provide important strategies for enhancing swine immunity and reducing the reliance on antibiotics in pig production.

The spleen is the largest peripheral lymphoid organ in the body, harboring a large number of mature lymphocytes. Among splenic lymphocytes, approximately 55% are B cells and about 25% are T cells (14). Moreover, the spleen contains a variety of immune factors and serves as a central site for both cellular and humoral immune responses. It can rapidly respond to pathogen invasion through neurohumoral regulatory pathways (15). Previous studies have suggested that FLF can enhance immune function in animals to some extent, but the mechanisms remain unclear. At present, most of the research on FLF focuses on the intestinal tract and microorganisms (16, 17), while systematic investigations into spleen transcriptomic and metabolic alterations remain limited. Therefore, this study used transcriptomics and metabolomics methods to systematically analyze the spleen of pigs fed FLF, and revealed the regulatory mechanism of FLF on pig immune function through multi-omics joint analysis. This study aims to provide a theoretical basis for the scientific application and promotion of FLF and the implementation of resistance-free pig breeding strategy.

## 2 Materials and methods

### 2.1 Sample collection and preparation

A total of 64 healthy commercial crossbred boars (Duroc×Landrace×Yorkshire; 90 days 50.06 ± 3.75 kg, animals that had not received any antibiotics) were enrolled in a 60-day feeding trial. Animals were stratified by initial body weight and randomly allocated to two treatment groups, each comprising four pens with eight pigs per pen. The control group (CON) received the basal diet in dry pellet form, whereas the LFF group was provided fermented liquid feed prepared by mixing the basal diet with water (1:3, w/v) followed by daily batch fermentation. Diets were formulated according to NRC (2012) guidelines (Table 1). Feed was delivered using an automated centralized feeding system (Osborne, Shanghai, China) at 12:00 and 16:00, and pigs had unrestricted access to feed and water throughout the trial.

**Table 1:** Ingredients and nutrient contents of the experimental diets (dry matter basis).

Ingredients		Nutriention level	
Items	Content (%)	Items	Content (%)
Corn	76.22	Digestible energy (DE, Kcal/kg)	3,270
Soybean meal	13.7	Crude protein (CP)	13.5
Wheat bran	3.65	Ca	0.48
Soybean oil	2.12	P	0.47
Stone powder	0.7	Available phosphorous	0.24
Dicalcium phosphate	0.69	Lysine	0.79
Salt	0.35	Methionine	0.28
Premix^1^	2.5	Methionine+Cystine	0.52
L-lysine HCl	0.28	Threonine	0.52%
Methionine	0.05	Tryptophan	0.14%
Threonine	0.05		

^1^Per kilogram: VA 200000 IU, VD 20000 IU, VE 2000 IU, VK 50 mg, niacim 200 mg, D-pantothenic acid 2000 mg, VB9 30 mg, riboflavin 250 mg, VB1 200 mg, VB12 1,000 μg, choline 100 g, antioxidants 20,000 mg, Biotin 5 mg, VB6 100 mg, Fe (as ferrous sulfate) 10,000 mg. Zn (as zinc sulfate) 10,000 mg, Mn (as manganese sulfate) 1,000 mg, Se (as sodium selenite) 30 mg, I (aspotassium iodide) 50 mg.

At the conclusion of the experiment, six pigs per treatment (twelve total) were randomly selected for sampling. Pigs were fasted for 8 h and deprived of water for 2 h prior to slaughter, then euthanized by electrical stunning followed by exsanguination. Blood (10 mL) was collected, allowed to clot at 4 °C, and centrifuged at 2500 r/min for 15 min to obtain serum, which was stored at −20 °C. Spleen tissues were excised immediately after slaughter, trimmed into ~1 cm^3^ sections on ice, placed into sterile cryovials, and snap-frozen in liquid nitrogen. To reduce intra-organ variability, samples used for sequencing were prepared as homogenized composites derived from both the left and right regions of the spleen (Three spleen samples were collected from each pig).

### 2.2 Preparation of FLF and selection of fermentation process

Dry feed was mixed with the bacterial inoculum at a solid-to-liquid ratio of 1:3 to prepare the fermentation substrate. The inoculum consisted of a compound microbial preparation dominated by *Lactobacillus plantarum* and supplemented with *Saccharomyces cerevisiae*. This microbial consortium possesses well-characterized probiotic properties, including the ability to inhibit common pathogenic bacteria in pigs, and is widely used to enhance microbial inoculation efficiency during the preparation of fermented liquid feed. Prior to inoculation, the microbial agent was dissolved or activated in a small volume of warm water (30–35 °C) and then evenly sprayed onto or incorporated into the feed–water mixture. Mechanical stirring was used to ensure uniform distribution of the inoculum. The final concentration of lactic acid bacteria at inoculation was 1.0 × 10^7^ CFU/mL.

The mixture was transferred into fermentation containers (Shanghai Bailun Biological Technology Co., Ltd., Shanghai, China), which were filled to no more than 70% of their total volume to allow adequate headspace. Excess air was expelled prior to sealing to create a semi-anaerobic environment. Fermentation was carried out at 26 ± 0.5 °C for 24–72 h. During fermentation, samples were collected every 12–24 h from multiple depths and locations using sterile pipettes or sampling spoons to measure pH, odor, microbial counts, and the presence of mold contamination.

### 2.3 Measurement of immune factor levels

The serum samples were thawed at 4 °C prior to analysis. The concentrations of interleukin-6 (IL-6), tumor necrosis factor-*α* (TNF-α), complement C3, immunoglobulin A (IgA), and interferon-*γ* (IFN-γ) were measured using commercial ELISA kits (Beyotime, China) according to the manufacturer’s instructions. Each sample was assayed in triplicate, and absorbance was recorded at 450 nm using a microplate reader. Cytokine concentrations were calculated based on the standard curves generated for each assay.

### 2.4 RNA extraction, library construction and sequencing

Total RNA was extracted using the TRIzol reagent (Invitrogen, CA, USA) according to the manufacturer’s protocol. RNA purity and quantification were evaluated using the NanoDrop 2000 spectrophotometer (Thermo Scientific, USA). RNA integrity was assessed using the Agilent 2,100 Bioanalyzer (Agilent Technologies, Santa Clara, CA, USA). Then the libraries were constructed using VAHTS Universal V6 RNA-seq Library Prep Kit according to the manufacturer’s instructions. The transcriptome sequencing and analysis were conducted by OE Biotech Co., Ltd. (Shanghai, China).

The libraries were sequenced on an llumina Novaseq 6,000 platform and 150 bp paired-end reads were generated. About 47.9 M raw reads for each sample were generated. Raw reads of fastq format were firstly processed using fastp1 and the low quality reads were removed to obtain the clean reads. Then about 47.02 M clean reads for each sample were retained for subsequent analyses. The clean reads were mapped to the reference genome (https://ftp.ncbi.nlm.nih.gov/genomes/all/GCF/000/003/025/GCF_000003025.6_Sscrofa11.1/, Version Number: NCBI _ Sscrofa11.1) using HISAT22. FPKM3 of each gene was calculated and the read counts of each gene were obtained by HTSeq-count4. PCA analysis were performed using R (v 3.2.0) to evaluate the biological duplication of samples.

### 2.5 RNA-seq data processing

Differential expression analysis was performed using the DESeq2. *p* value < 0.05 and |log2foldchange| > 1 was set as the threshold for significantly differential expression gene (DEGs). Hierarchical cluster analysis of DEGs was performed using R (v 3.2.0) to demonstrate the expression pattern of genes in different groups and samples. The radar map of top 30 genes was drew to show the expression of up-regulated or down-regulated DEGs using R packet ggradar. Based on the hypergeometric distribution, GO (http://geneontology.org/), KEGG pathway (http://www.genome.jp/kegg/), Reactome and WikiPathways enrichment analysis of DEGs were performed to screen the significant enriched term using R (v 3.2.0), respectively. R (v 3.2.0) was used to draw the column diagram, the chord diagram and bubble diagram of the significant enrichment term.

### 2.6 LC–MS/MS analysis

A total of 30 mg of each sample was transferred to a 1.5mL EP tube. After adding 600 μL extract (containing L-2-chlorophenylalanine, 4 μg/mL), the sample was vortexed for 30 s, ultrasonically treated in an ice water bath for 10 min, and incubated for 1 h to precipitate the protein. The sample was then centrifuged at 12,000 r / min for 15 min. The supernatant was transferred to a new glass vial for analysis. Quality control (QC) samples were prepared by mixing equal amounts of supernatants from all samples.

LC–MS / MS analysis was performed using ACQUITY UPLC I-Class plus ultra-high performance liquid chromatography tandem QE high-resolution mass spectrometer: Chromatographic column: ACQUITY UPLC HSS T3 (100 mm × 2.1 mm, 1.8 um); column temperature: 45 °C; mobile phase: A-water (containing 0.1% formic acid), B-acetonitrile (containing 0.1% formic acid); flow rate: 0.35 mL / min; injection volume: 2 μL. Mass spectrometry conditions: ion source: ESI; sample mass spectrometry signal acquisition was performed in positive and negative ion scanning modes, respectively.

### 2.7 Metabolomics analysis

Raw metabolomics data were processed using Progenesis QI v3.0 (Nonlinear Dynamics, Newcastle, UK). Preprocessing steps included baseline correction, peak detection, peak integration, retention time alignment, and data normalization. Mass-matching tolerances were set to 5 ppm for precursor ions when referencing HMDB and Lipidmaps, and 10 ppm for the LuMet-Animal and METLIN databases; fragment ion tolerances were set to 10 ppm and 20 ppm, respectively. Metabolite annotation integrated multiple orthogonal features, including retention time (RT), accurate mass, MS/MS fragmentation patterns, and isotope distributions, and was performed against the Human Metabolome Database (HMDB), Lipidmaps (v2.3), METLIN, and the LuMet-Animal 3.0 local database.

Processed data were subsequently subjected to quality control procedures, including missing-value filtering, zero-value replacement, score-based filtering, and data merging. Ion features with mean missing (or zero) values exceeding 50% within any treatment group were removed. Remaining zero values were imputed with half of the minimum non-zero signal intensity across all samples (non-imputed values are documented in the accompanying missing-value matrix). Candidate metabolites were further filtered according to annotation scores generated by Progenesis QI. Annotation confidence was categorized into four levels (Level 1: RT ± 0.3 min and fragmentation score ≥ 45; Level 2: RT ± 0.3 min and fragmentation score < 45; Level 3: fragmentation score ≥ 45; Level 4: fragmentation score < 45). A threshold score of 36 (out of 80) was applied, and metabolites below this cutoff were excluded as unreliable identifications.

Multivariate statistical analyses were conducted using SIMCA 14.1 (Umetrics, Sweden). Principal component analysis (PCA) was first employed to examine sample clustering, distribution homogeneity, and potential outliers. Partial least squares discriminant analysis (PLS-DA) and orthogonal PLS-DA (OPLS-DA) were subsequently performed to identify discriminatory metabolic features between groups. Model performance was evaluated using R^2^X, R^2^Y, and Q^2^ statistics, and model robustness was verified through 200-fold permutation testing. Variable importance in projection (VIP) scores were used to estimate the contribution of each metabolite to the discriminant model. Differential metabolites were defined as those with VIP > 1.0 and a false discovery rate *P*-adj < 0.05. Pathway enrichment analyses were conducted using the KEGG database (https://www.genome.jp/kegg/) via the MetaboAnalyst platform, and pathway significance and impact values were determined (*p* < 0.05).

### 2.8 qPCR

To validate the accuracy of the sequencing results, a subset of differentially expressed genes was selected for quantitative real-time PCR (qRT-PCR) analysis (Each sample was analyzed in three technical replicate wells for PCR detection.). Total RNA was reverse-transcribed into cDNA using a reverse transcription kit (TIANGEN) according to the manufacturer’s instructions. qRT-PCR was performed on a Bio-Rad CFX96 Real-Time Detection System (Bio-Rad, Hercules, CA, USA) using the SYBR Premix Ex Taq kit (TaKaRa). Each 20 μL reaction contained 9 μL of SYBR Premix, 2 μL of cDNA, 1 μL each of forward and reverse primers, and 7 μL of ddH₂O. The amplification protocol consisted of an initial denaturation at 95 °C for 5 s, followed by 40 cycles of 95 °C for 5 s and 60 °C for 30 s, with a final step of 65 °C for 5 s and 95 °C for 5 s. Relative gene expression levels were calculated using the 2^-ΔΔCt^ method with GAPDH as the internal control. Primer sequences are listed in Supplementary Table S1.

### 2.9 Integrated analysis of metabolomics and transcriptomics

Transcriptome and metabolome joint analysis was performed using R 3.2 and Python 3.9. First, differentially expressed genes (DEGs) and differentially expressed metabolites (DEMs) underwent separate normalization processes. DEGs underwent log₂ transformation based on FPKM values and low-expression genes were filtered out; DEMs underwent log₁₀ transformation based on peak area and Pareto scaling to reduce the impact of outliers. The mixOmics package (v6.20) was used to construct a cross-omics association model based on sparse partial least squares (sPLS). Outputs included a cross-omics correlation matrix and a weighted correlation network. Subsequently, a gene–metabolite correlation network was constructed. Spearman correlation coefficients were calculated for all DEGs and DEMs, retaining significant strong correlations (*P*-adj< 0.05 and |R| > 0.7). Select DEGs and DEMs with|R| > 0.8 for network visualization using Cytoscape (v3.9.0). Key nodes were identified based on degree centrality and betweenness centrality. Combining joint pathway enrichment analysis, MetaboAnalyst v5.0 was employed for metabolite enrichment in KEGG pathways, while clusterProfiler (v4.6) performed KEGG enrichment for DEGs. Significant pathways (*p* < 0.05) from both analyses were then filtered.

### 2.10 Statistical analysis

Qpcr and growth performance data data were statistically analyzed using GraphPad Prism software (v9.5). The difference between the two groups was evaluated by Student’s *t* test, and the results were expressed as mean ± standard deviation (mean ± SD). When *p* < 0.05, the difference was considered statistically significant.

## 3 Result

### 3.1 Effects of FLF on growth performance and immune factor levels in pigs

**Figure 1:**
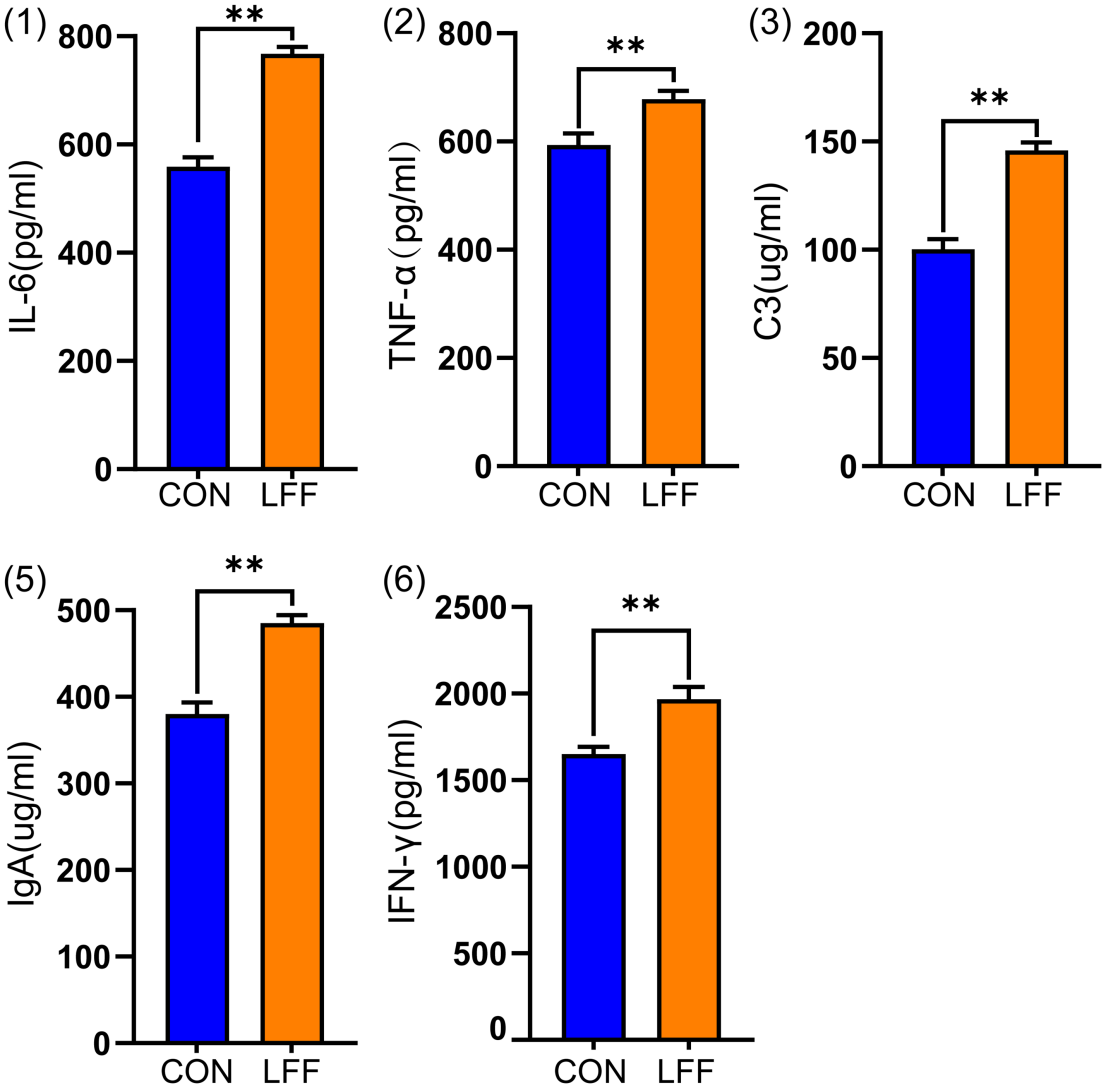
Bar graph showing differences in spleen immune factor levels between the CON and LFF groups. CON, control group fed a normal diet, represented by blue bars; LFF, group fed fermented liquid feed, represented by orange bars. *indicates *p* < 0.05; **indicates *p* < 0.01.

During the whole experimental period, feeding fermented liquid feed (LFF) significantly improved the growth performance of pigs. Compared with the control group (CON), pigs in the LFF group had higher body weight at the end of the experiment and showed a significant increase in average daily gain and FCR (Table 2). In addition, the results of blood immunological indexes showed that the levels of serum IL-6, TNF-*α*, C3, IgA and IFN-*γ* in LFF group were significantly higher than those in CON group (Figure 1A), suggesting that fermented liquid feed could promote the growth and development of pigs and modulate the immune function of pigs.

**Table 2:** The growth parameters of pigs.

Term	CON	LFF	*p*-value
Phase one (1–30 days)			
Primary weight (Kg)	50.07 ± 3.64	50.06 ± 4.2	>0.05
Final weight (Kg)	73.92 ± 3.3	78.50 ± 3.73	<0.05
Phase two (31–60 days)			
Primary weight (Kg)	73.92 ± 3.3	78.50 ± 3.73	<0.05
Final weight (Kg)	102.65 ± 3.46	109.92 ± 3.73	<0.05
The entire experimental period (1–60 days)			
Primary weight (Kg)	50.07 ± 3.64	50.06 ± 4.2	>0.05
Final weight (Kg)	102.65 ± 3.46	109.92 ± 3.73	<0.05
Weight gain (Kg)	52.58 ± 1.6	57.86 ± 2.21	<0.01
Average daily gain (Kg)	0.88 ± 0.03	0.96 ± 0.04	<0.01
Average daily feed intake (g)	2446 ± 183.71	2745 ± 190.23	<0.05
Feed conversion ratio	2.78 ± 0.04	2.89 ± 0.02	<0.01

### 3.2 Overview of spleen transcriptome profiles in CON and LFF groups

In this study, a total of 12 cDNA libraries (CON-1 to CON-6 and LFF-1 to LFF-6) were constructed from spleen samples of the CON and LFF groups and subjected to high-throughput sequencing on the Illumina platform. On average, about 47.88 million and 47.93 million raw reads were obtained in the CON and LFF groups, respectively, and the quality assessment showed that the average GC content of the samples in the two groups exceeded 50% and the Q30 value was about 98%, which indicated that the sequencing data were highly reliable. After removing low-quality and redundant sequences, an average of 46.97 million and 47.06 million clean reads were obtained in the CON and LFF groups, respectively. Subsequently, HISAT2 (v2.2.1, http://github.com/infphilo/hisat2) was utilized to align the clean reads to the pig reference genome, and the alignment rate ranged from 91.89–93.77% (Supplementary Table S2). In conclusion, the quality of RNA sequencing data obtained in this study is reliable and can be used for subsequent bioinformatics analysis.

### 3.3 Identification and analysis of differentially expressed genes between CON and LFF groups

**Figure 2:**
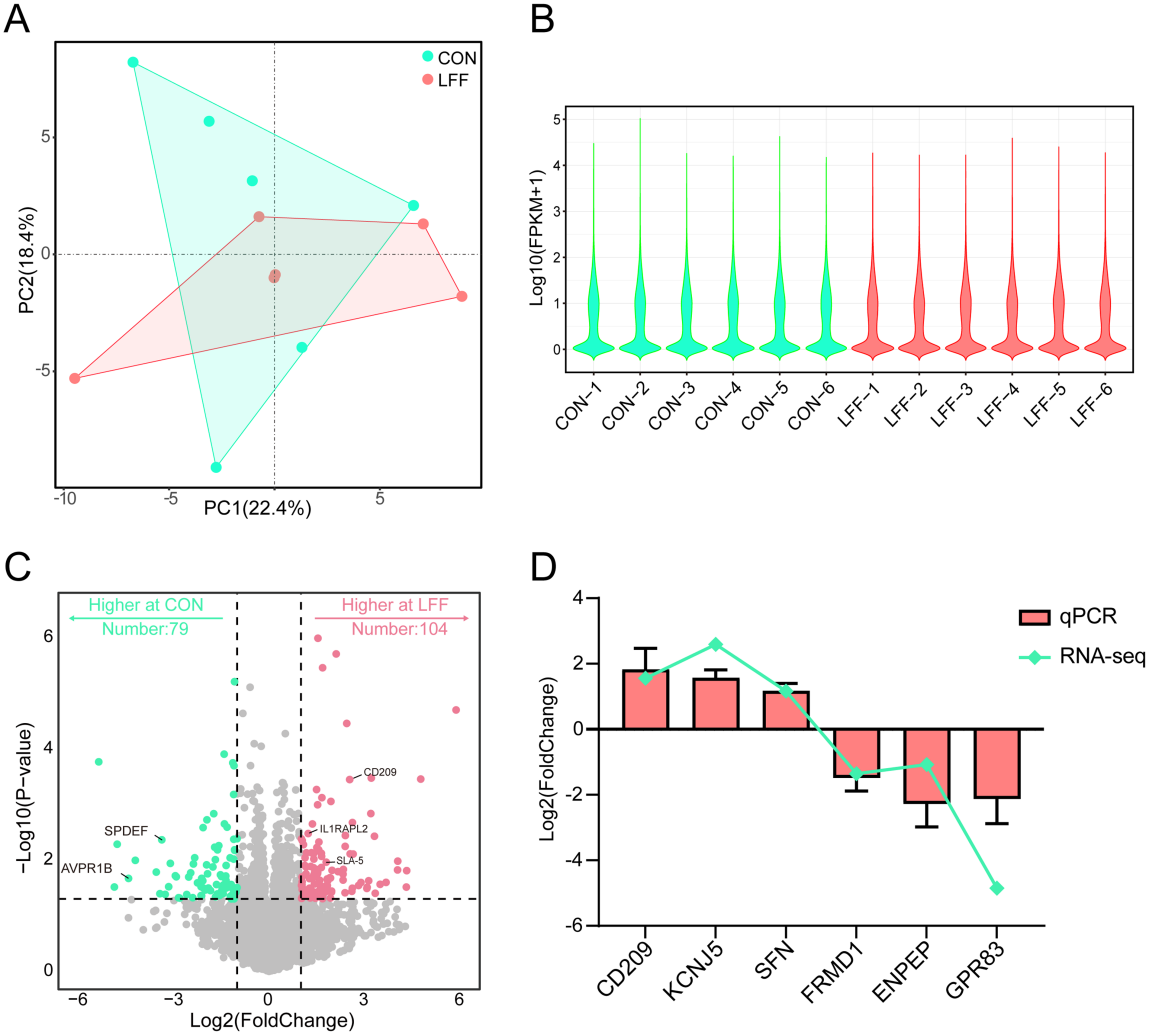
Differential analysis of spleen transcriptomes between CON and LFF groups. **(A)** PCA analysis plot of CON and LFF group samples, where light blue represents the solid diet group and pink indicates the FLF group; **(B)** Boxplot of FPKM values for CON and LFF group transcriptomes, where light blue represents the solid diet group and pink indicates the FLF group; **(C)** Volcano plot of differentially expressed transcripts in spleen between CON and LFF groups. Light blue indicates genes expressed in the solid diet group; pink indicates genes expressed in the LFF group; **(D)** qPCR validation of expression trends for differentially expressed genes between CON and LFF groups. Vertical axis: log_2_FC; horizontal axis: gene name. Pink bars indicate log_2_FC values from qPCR results in both groups; light blue lines indicate log_2_FC values from RNA-seq data in both groups. FPKM, fragments per kilobase per million; CON, control group fed normal diet; LFF, fermented liquid feed group.

Principal component analysis (PCA) was performed based on the spleen transcriptomic profiles obtained from the CON and LFF groups. The first two principal components, PC1 and PC2, explained 22.38% and 17.08% of the total variance, respectively, and clearly distinguished the CON group from the LFF group (Figures 2A,B). This indicates that fermented liquid feed exerts a notable influence on the spleen transcriptome of pigs.Gene expression levels were then normalized using fragments per kilobase of transcript per million mapped reads (FPKM). The overall gene expression patterns between the two groups were found to be largely similar, as shown by the FPKM distribution plot.Differentially expressed genes (DEGs) were subsequently identified using the DESeq2 package (https://bioconductor.org/packages/release/bioc/html/DESeq2.html). A total of 184 DEGs were detected based on the criteria of |log₂ fold change| > 1 and *p* < 0.05, including 104 upregulated and 79 downregulated genes in the LFF group (Figure 2C). To validate the RNA-seq results, 12 DEGs were randomly selected for RT-qPCR analysis. The expression patterns of these genes were highly consistent with the RNA-seq data (Figure 2D), confirming the reliability and accuracy of the transcriptomic analysis.

### 3.4 Functional enrichment analysis of differentially expressed genes between CON and LFF groups

**Figure 3:**
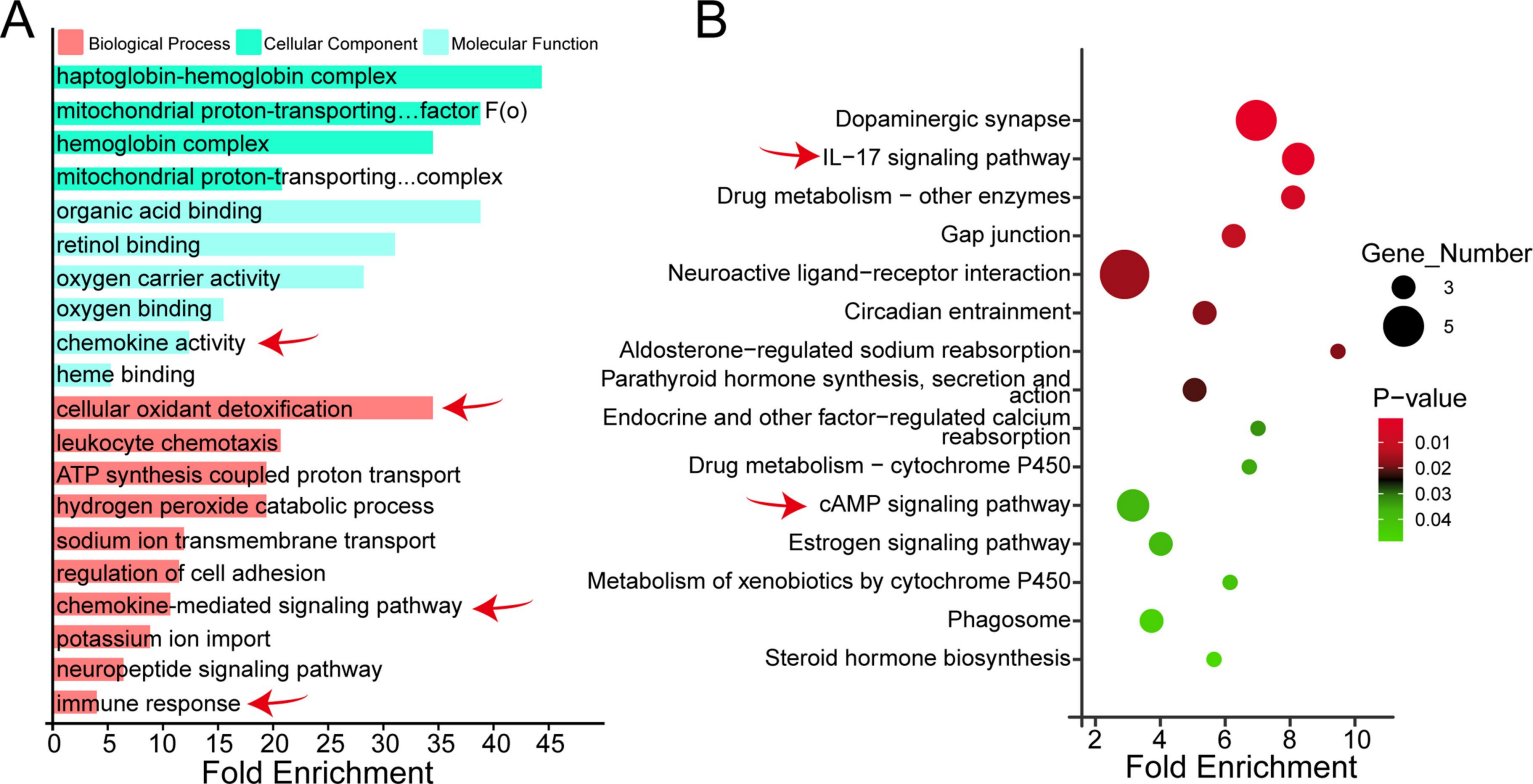
Functional enrichment analysis of differential genes between CON and LFF groups. **(A)** GO enrichment analysis classification histogram of differentially expressed genes between the CON and LFF groups, showing significantly enriched GO terms; **(B)** KEGG enrichment analysis bubble chart of differentially expressed genes between the CON and LFF groups, showing significantly enriched KEGG items. CON, control group (normal feed); LFF, liquid fermented feed group.

To further explore the functions of differentially expressed genes (DEGs), gene ontology (GO) enrichment analysis was performed on 187 DEGs. The results showed that these DEGs were annotated to a total of 866 GO entries, including 232 biological process (BP), 88 cellular component (CC), and 113 molecular function (MF) entries. A total of 63 entries reached the significant level at a set threshold of *p* < 0.05, and the top 20 significantly enriched GO entries are shown in Figure 3A. Specifically, there were 31 significantly enriched entries in the BP category, involving cellular oxidant detoxification, leukocyte chemotaxis, neuropeptide signaling pathway and regulation of cell adhesion; Thirteen entries were significantly enriched in the CC category, including hemoglobin complex, haptoglobin-hemoglobin complex, and sodium channel complex; 19 entries were significantly enriched in the MF category, mainly involving chemokine Of the 31 notable entries in the BP category, many are closely related to immunity, such as immune response, chemokine-mediated signaling pathway, neurotrophic pathway, and the immune response. Further analysis showed that genes such as *CXCL2*, *CXCL*8, *OSM* and *SLA-5* were closely associated with the immune response (Figure 3A).

KEGG pathway annotation was performed for the identified differentially expressed genes (DEGs). A total of 150 KEGG pathways were annotated (Figure 3B), with 27 pathways significantly enriched at *p* < 0.05. For instance, pathways including Dopaminergic synapse, Phagosome, Drug metabolism-cytochrome P450, and Parathyroid hormone synthesis, secretion and action were significantly enriched. Multiple genes were enriched in immune-related pathway entries, including the IL-17 signaling pathway, cAMP signaling pathway, and Aldosterone-regulated sodium reabsorption. Further analysis indicated that genes such as *CXCL2*, *CXCL8*, *FOS*, and *FOSB* were enriched in these immune-related pathways, suggesting their potential involvement in the regulation of host immune responses. KEGG pathway annotation was performed for the identified differentially expressed genes (DEGs).

### 3.5 Alterations in spleen metabolic profiles between CON and LFF groups

**Figure 4:**
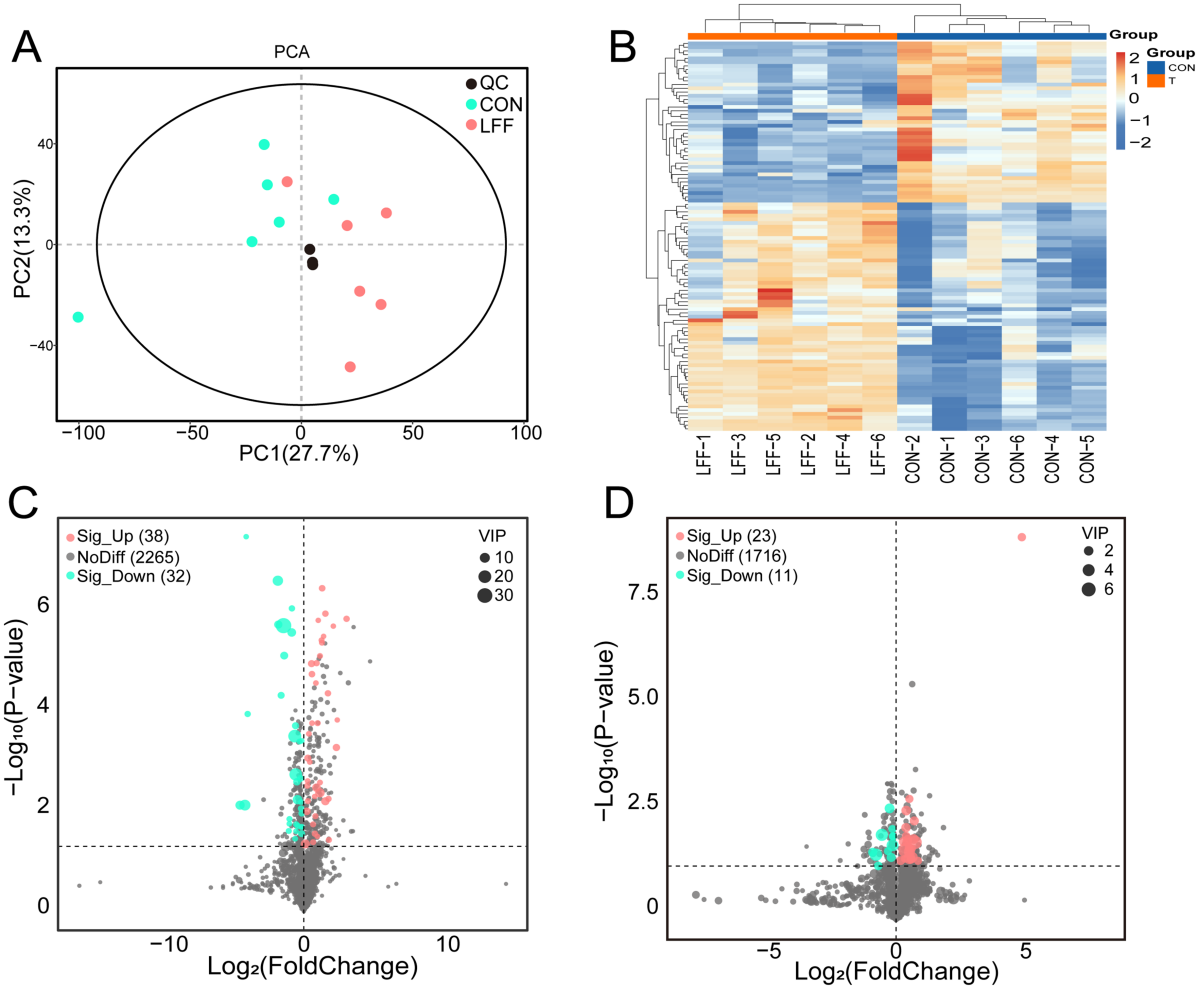
Analysis of spleen metabolome differences between CON and LFF groups. **(A)** PCA of metabolite differences between the normal group and the fermented liquid group. Pink represents the FLF-fed group, light blue represents the CON group, and black represents QC; **(B)** Heatmap of metabolite abundance differences between the normal group and the fermented liquid group. Blue represents the CON group, orange represents the FLF-fed group; **(C)** Volcano plot of metabolite differences in cation patterns between the normal group and the fermented liquid group. Pink indicates metabolites significantly up-regulated in the FLF group, light blue indicates metabolites significantly up-regulated in the CON group relative to the FLF group; **(D)** Volcano plot of anion pattern differences between the normal group and the fermented feed group, pink indicates metabolites significantly up-regulated in the FLF group, light blue indicates metabolites significantly up-regulated in the CON group relative to the FLF group. CON, normal feed group; LFF, fermented feed group.

Immune factor detection results revealed significant differences in serum immune factor levels between the CON and LFF groups, suggesting that feeding liquid fermented feed may influence metabolic patterns in pigs. To investigate this, we conducted metabolomics analysis on spleen samples from both groups using a high-resolution LC-MS platform. A total of 1,750 and 2,335 metabolites were identified in anion and ion modes, respectively. OPLS-DA analysis revealed that the two principal components, PC1 and PC2, explained 27.7% and 13.3% of the total variance, respectively. In the score plot, samples from the CON group and LFF group exhibited distinctly separate distribution regions, indicating that the two groups possessed clearly distinguishable metabolomic profiles (Figure 4A). Heatmap analysis further highlighted differences in metabolite abundance patterns between groups (Figure 4B).Based on this, differential metabolites were identified by setting screening thresholds: VIP > 1, |log2Fold Change| > 1, and *p < 0.05*. Results showed that in the anion mode, 34 differential metabolites were identified, including 23 significantly upregulated and 11 significantly downregulated; while 70 differentially expressed metabolites were identified in the cationic mode, comprising 38 significantly upregulated and 32 significantly downregulated metabolites (Figures 4C,D). Collectively, these findings demonstrate significant differences in the metabolic profiles between the CON and LFF groups, providing metabolomic support for the hypothesis that fermented liquid feed exerts regulatory effects on porcine physiological functions.

### 3.6 Functional analysis of differential metabolites

**Figure 5:**
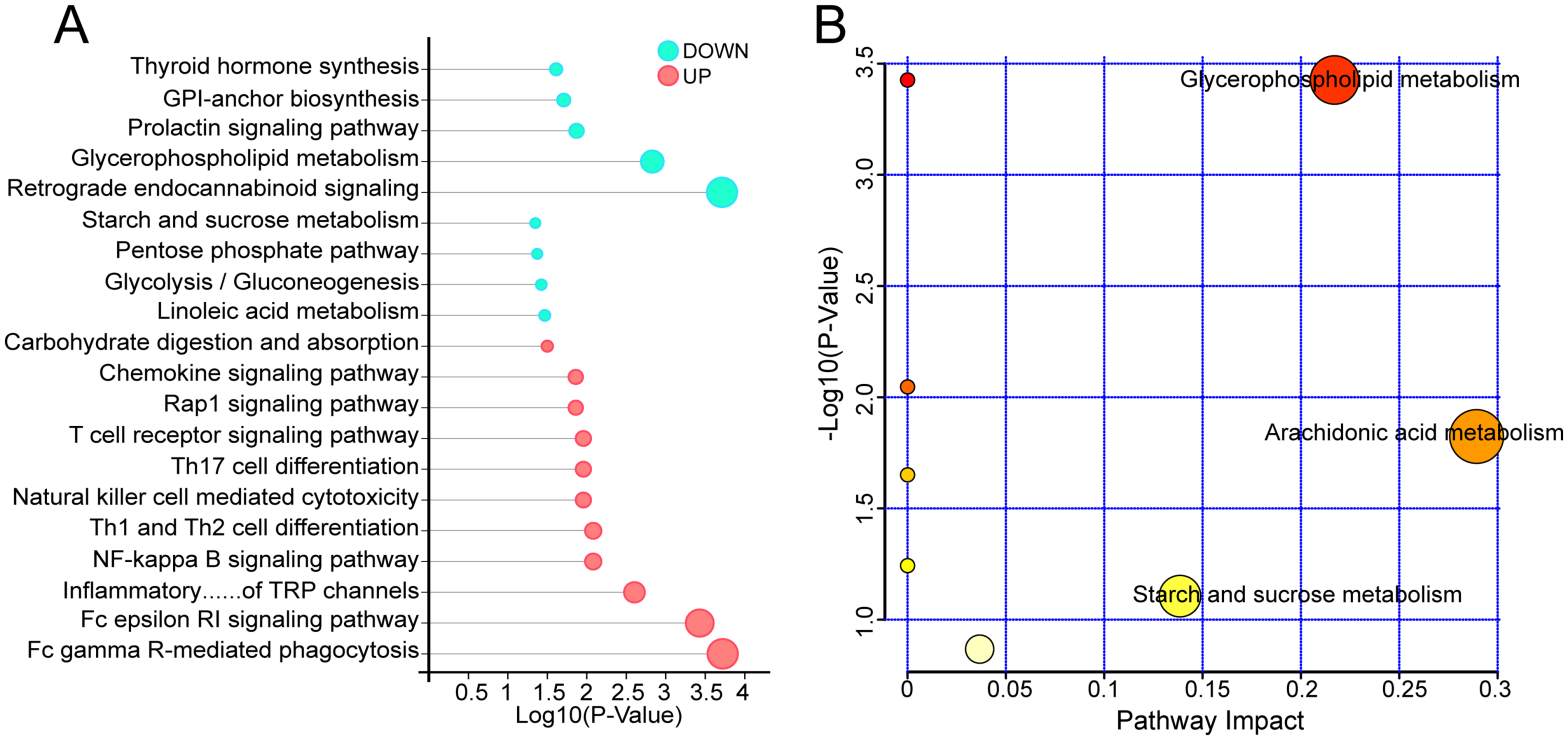
KEGG enrichment analysis of differential metabolites in the spleen of CON and LFF groups. **(A)** KEGG analysis bubble chart of differentially expressed metabolites between the normal diet group and the fermented liquid feed group. Pink bubbles indicate significantly upregulated KEGG entries, while light blue bubbles indicate significantly downregulated KEGG entries. **(B)** Pathway impact analysis bubble chart of differentially expressed metabolites. Pathways closer to the upper right corner indicate more significant pathway effects. CON, normal diet group; LFF, fermented liquid feed group.

To explore the biological processes potentially involved in differential expression metabolites (DEMs) *in vivo*, KEGG enrichment analysis was performed. Results showed that a total of 96 KEGG pathways were annotated, with 71 significantly enriched at the *p* < 0.05 threshold (Figure 5A). Significantly enriched pathways primarily involved Biosynthesis of unsaturated fatty acids, Insulin secretion, Linoleic acid metabolism, Glycerophospholipid metabolism, Aldosterone synthesis and secretion, and Regulation of lipolysis in adipocytes.Notably, multiple immune-related signaling pathways were also significantly enriched, including NF-kappa B signaling pathway, Th1 and Th2 cell differentiation, Th17 cell differentiation, Chemokine signaling pathway, T cell receptor signaling pathway, and B cell receptor signaling pathway. Further pathway impact analysis revealed significant effects on Arachidonic acid metabolism and Glycerophospholipid metabolism (Figure 5B), suggesting these metabolic pathways may play a crucial role in LFF’s regulation of immunity and metabolism.

### 3.7 Integrative analysis of transcriptomics and metabolomics data

**Figure 6:**
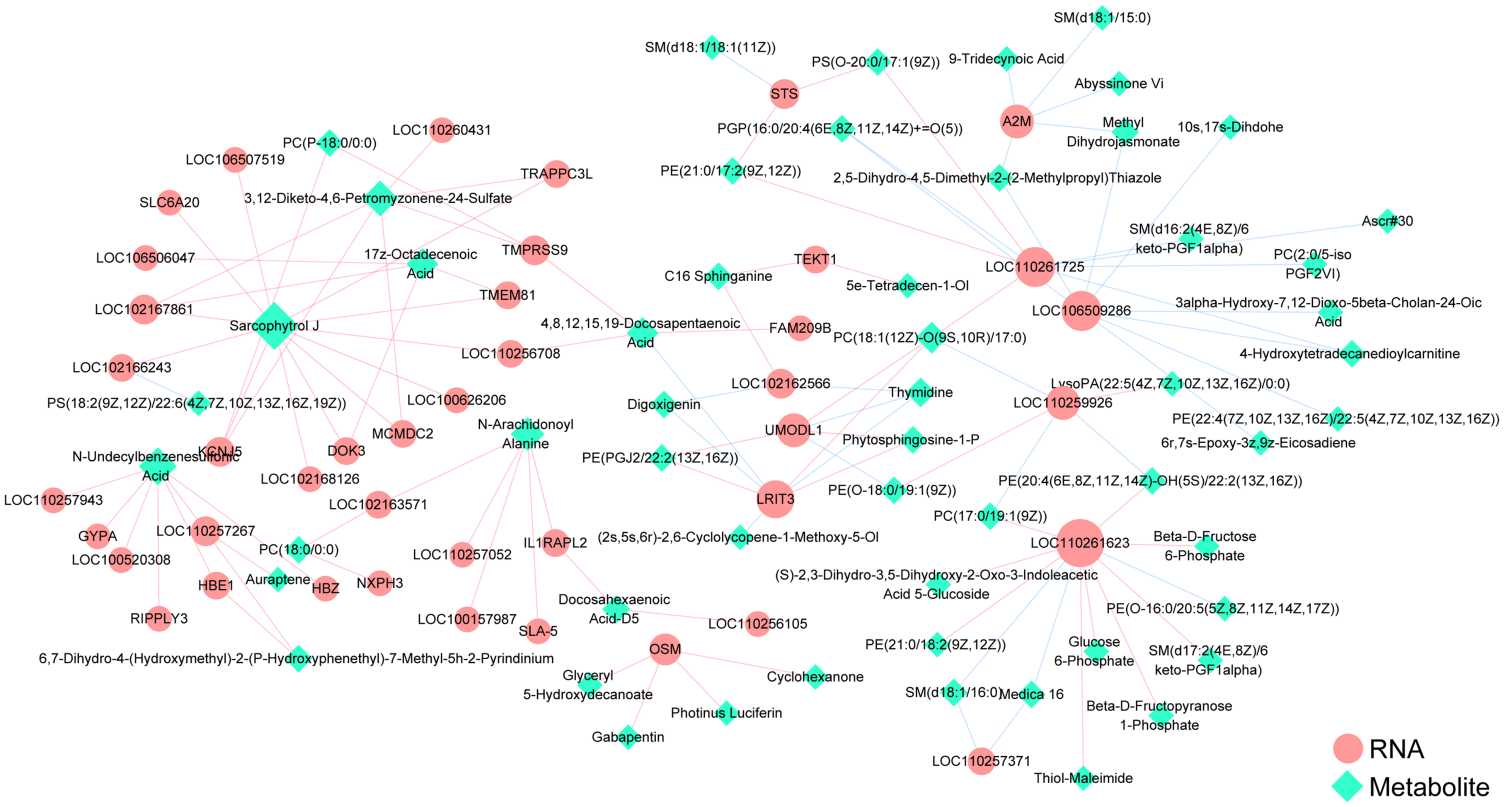
Network diagram of joint analysis of differential metabolites and differentially expressed genes in spleens between the CON and LFF groups. Red circles indicate differentially expressed genes in the transcriptome; green circles indicate differentially metabolized metabolites in the metabolome; circle size represents degree values; blue lines indicate negative correlations between DEM and DEG; red lines indicate positive correlations between DEM and DEG. CON, normal feed group; LFF, fermented liquid feed group.

To further elucidate the relationship between transcriptomes and metabolomes, a joint analysis of differentially expressed genes (DEGs) and metabolites (DEMs) in the CON and LFF groups was conducted, establishing a gene-metabolite correlation network. The analysis indicates that fermented liquid feed may induce immune-related responses by regulating transcriptional levels and metabolic processes within the body. This network comprises 48 differentially expressed genes and 61 differential expression patterns, with 116 pairs of correlation relationships. Among these, SLC6A20, IL1RAPL2, SLA-5, OSM, and FAM209B exhibited significant positive correlations with Sarcophytrol J, N-Arachidonoyl Alanine, Glyceryl 5-Hydroxydecanoate, Gabapentin, Photinus Luciferin, Cyclohexanone, 4,8,12,15,19 -Docosapentaenoic Acid. Conversely, A2M exhibited negative correlations with 5-Dihydro-4,5-Dimethyl-2-(2-Methylpropyl)Thiazole, Methyl Dihydrojasmonate, 9-Tridecynoic Acid, and SM(d18:1/15:0). This suggests that FLF may influence the metabolic patterns of the host (Figure 6).

## 4 Discussion

Although swine diets tend to be nutritionally balanced, they inevitably contain certain antinutritional factors. For instance, soybean meal commonly contains high levels of protease inhibitors, β-conglycins, and phytate. These components limit the absorption of nutrients in the animal’s gut, thereby reducing feed utilization efficiency (18, 19). Fermentation is recognized as an economical and effective method for enhancing the nutritional quality and functional properties of feed ingredients. For instance, co-fermentation using *Bacillus subtilis*, *Saccharomyces cerevisiae*, *Lactobacillus plantarum*, and phytase significantly increases crude protein, trichloroacetic acid-soluble protein, soluble dietary fiber, and lactic acid levels in diets. Simultaneously, it reduces antinutritional factors and insoluble dietary fiber content, enhancing feed digestion and absorption capacity while promoting growth performance (20). A study by Cheng et al. further demonstrated that replacing soybean meal with fermented compound protein improved growth performance, nutrient digestibility, and carcass traits in growing-finishing pigs, without exerting any adverse effects on meat quality or animal health (21). In this study, although the molecular mechanisms by which fermented liquid feed improves growth performance remain unelucidated, our data provide evidence of its impact on porcine immune function. With the implementation of the “antibiotic ban” policy and its gradual emergence as a trend in the livestock industry (1), exploring alternatives to antibiotics holds significant importance. Our result found that feeding fermented liquid feed significantly increased the levels of IgA, TNF-*α*, IL-6, IFN-*γ*, and C3 in pig blood. This indicates that FLF exerts a positive effect on promoting the body’s immune function.

Previous studies have demonstrated that fermented liquid feed can improve animal health (22, 23). For example, feeding fermented liquid feed has been shown to alleviate weaning-induced intestinal stress in piglets (24). In this study, RNA-seq analysis revealed a large number of differentially expressed genes between the spleen tissues of the CON group and the LFF group. Among these, multiple immune-related genes were significantly upregulated in the LFF group, such as *CXCL8*, *OSM*, and *SLA-5*. Functional enrichment analysis further indicated that these differentially expressed genes were primarily involved in immune-related pathways, including the IL-17 signaling pathway, cAMP signaling pathway, and aldosterone-regulated sodium reabsorption. Notably, genes such as *CXCL2*, *CXCL8*, *OSM*, *SLA-5*, *FOS*, and *FOSB* played key roles within these pathways. *CXCL2* and *CXCL8* belong to the chemokine superfamily, and the proteins they encode play critical roles in immune regulation and inflammatory responses (25, 26). Furthermore, we compared the metabolic profiles of the spleen between the CON and LFF groups. The results showed that feeding with fermented liquid feed induced a more pronounced metabolic reprogramming, primarily involving pathways related to immune metabolism and fatty acid synthesis. Specifically, the NF-κB signaling pathway, Th17 cell differentiation, chemokine signaling pathway, biosynthesis of unsaturated fatty acids, and arachidonic acid metabolism were significantly enriched in the LFF group. Meanwhile, the abundances of metabolites such as arachidonic acid, glucose-6-phosphate, and stearic acid were significantly increased in the LFF group. Arachidonic acid (AA), a representative polyunsaturated long-chain fatty acid, is critically involved in the regulation of inflammation and immune function (27, 28). AA and its derivatives can also regulate the intensity and duration of inflammation by balancing pro-inflammatory and anti-inflammatory effects (29, 30). Glucose-6-phosphate (G6P) is an important intermediate product of the glycolysis pathway (31, 32), Previous studies have shown that the abundance of G6P decreased significantly in PK-15 cells infected with classical swine fever virus, suggesting that it may be involved in the antiviral defense process of pigs (33).

In the study, we further conducted a joint analysis of DEGs and DEMs and constructed a correlation network. This network comprised 48 DEGs and 61 DEMs. Multiple immune-related genes exhibited strong positive correlations with differential metabolites, such as SLC6A20 with Sarcophytrol J, IL1RAPL2 with Docosahexaenoic Acid-D5, SLA-5 with N-Arachidonoyl Alanine, and OSM with Glyceryl 5-Hydroxydecanoate. Furthermore, we identified significant negative correlations between A2M and 2,5-Dihydro-4,5-Dimethyl-2-(2-Methylpropyl)Thiazole, Methyl Dihydrojasmonate, 9-Tridecynoic Acid, and SM(d18:1/15:0). Differential expression analysis revealed that FAM209B, IL1RAPL2, SLA-5, and SLC6A20 were upregulated in the LFF group. These genes are implicated in key immune processes, including cytokine and chemokine secretion (34), antiviral defense (35), and immune recognition (36), suggesting that their elevated expression may be associated with enhanced immune responsiveness (34, 37, 38). In contrast, A2M and OSM were upregulated in the CON group. A2M is a widely recognized prognostic biomarker, with higher expression often associated with adverse clinical outcomes (39). OSM, a pleiotropic cytokine involved in multiple inflammatory responses (40), has been identified as a contributing factor in several inflammatory diseases, including inflammatory bowel disease (41), cutaneous inflammatory disorders (42), and liver diseases (43).

## 5 Conclusion

In this study, we found that feeding FLF improved growth performance, elevated immune factor levels, and altered the transcriptomic and metabolomic profiles of the porcine spleen. Integrated multi-omics analysis revealed that FLF may enhance immune function by modulating the expression of spleen immune-related genes and reshaping metabolic pathways. However, further experimental validation is still required to substantiate these findings.

## Data Availability

The datasets presented in this study can be found in online repositories. The names of the repository/repositories and accession number(s) can found in NCBI BioProject PRJNA1236517/Supplementary material. Further inquiries can be directed to the corresponding author.
